# Algal Growth Regulators: Releasing Plant Hormones for Sustainable Horticulture

**DOI:** 10.3390/plants15091397

**Published:** 2026-05-02

**Authors:** Ibtissem Ben Hammouda, Katarzyna Pokajewicz, Beata Messyasz, Bogusława Łęska, Radosław Pankiewicz, Piotr P. Wieczorek

**Affiliations:** 1 Faculty of Chemistry and Pharmacy, Department of Analytical Chemistry, Opole University, Oleska 48, 45-052 Opole, Poland; ibtissem.benhammouda@uni.opole.pl (I.B.H.); katarzyna.pokajewicz@uni.opole.pl (K.P.); 2 Faculty of Biology, Adam Mickiewicz University in Poznań, Uniwersytetu Poznańskiego 6, 61-614 Poznań, Poland; beata.messyasz@amu.edu.pl; 3 Faculty of Chemistry, Adam Mickiewicz University in Poznań, Uniwersytetu Poznańskiego 8, 61-614 Poznań, Poland; boguslawa.leska@amu.edu.pl (B.Ł.); radpan@amu.edu.pl (R.P.)

**Keywords:** phytohormones, macroalgae, plant, microalgae, bioregulators, biostimulants, biofertilizers, UPLC-MS/MS method, QuEChERS

## Abstract

Phytohormones, or plant hormones, are intrinsic organic compounds within plants. These compounds have a significant impact as essential plant growth and development regulators, influencing processes from seed germination to fruit ripening. The exogenous application of these phytohormones, such as gibberellic acid (GA_3_), indole-3-acetic acid (IAA), and brassinosteroids, has been shown to significantly enhance horticultural productivity, with reported increases in germination, growth, and yield ranging from 10–40%. These signaling molecules are also vital for micro and macroalgae development and functioning. Recognizing their presence within algae presents a fresh perspective for horticultural researchers and cultivators, offering opportunities to enhance the quality and application of horticultural crops. Nevertheless, the challenge arises from the presence of phytohormones in trace amounts, complicating their extraction and identification. This paper will offer a comprehensive overview of phytohormone classification and detection methods and highlight their presence in algae, which may serve as an alternative for promoting plant growth in agriculture.

## 1. Introduction

As technology and society progress, horticultural crops are emerging as more than just a means of economic sustenance; they are also becoming increasingly significant in shaping human life [1]. The demand for consuming plants (such as vegetables and fruits) rises due to their vital role in the healthy human diet [2]. Such foods are rich in dietary fibers, vitamins, antioxidants, and minerals [3]. Hence, the global production of vegetables, for instance, surged by almost 66% in 2020, rising from 447 to 1130 million tons [4]. This highlights the significant effort required to improve sustainable agricultural crop production and address nutritional issues in the face of changing climate conditions [5,6]. Stressful conditions threaten the worldwide cultivation of crops [6]. These challenges encompass abiotic stress factors like drought, salinity, temperature fluctuations, humidity, intense light exposure, ultraviolet radiation, mineral deficiencies, heavy metals, and biotic stressors, including insects, pests, and diseases [7,8,9,10]. Drought and salinity are particularly damaging conditions, significantly impacting plants’ growth, developmental stages, and yield [11]. Numerous researchers recommend adopting sustainable management practices to enhance vegetable production under these stressful conditions [6,12,13]. Researchers are exploring the role of phytohormones due to their varied properties in combating stressful environmental conditions [6,7,13,14,15,16].

Phytohormones are key targets for effective regulation of the development and senescence of agricultural products [16,17,18], providing a mechanism to circumvent stress at different morphological, physiological, and molecular levels [19,20]. These compounds are a group of signal molecules produced in plants and have significant effects on metabolism, even at low concentrations [21,22].

Abundant in higher plants and playing a crucial role in crop development and yield [16,19], phytohormones are also present in macro- and microalgae [23,24]. Algae have been integrated into various agricultural systems worldwide and extensively examined as biofertilizers and biostimulants [23].

## 2. Phytohormones in Plant Development and Yield Regulation

**Figure 1:**
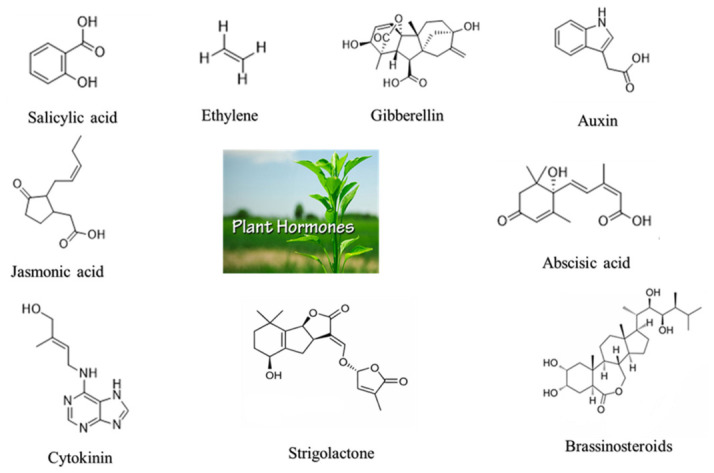
Structures of different phytohormones.

Phytohormones play an essential role in the regulation of functions in plants. These compounds regulate fundamental processes such as cell division, elongation, differentiation, morphogenesis, and metabolism [16]. Traditionally recognized plant hormones include auxins, which control root growth and development [21,24,25]; cytokinins, which promote cell division, organ formation, and delay aging [26,27]; abscisic acid (ABA), a key stress hormone that helps plants tolerate drought and other stresses [28,29,30]; gibberellins, which stimulate stem elongation, seed germination, and stress tolerance [31,32]; and ethylene, a gaseous hormone involved in fruit ripening and stress responses [33,34] (Figure 1). More recently, brassinosteroids [35], jasmonates [36], salicylic acid [37], and strigolactones [38] were identified and recognized as non-classical plant hormones [16,23].

**Figure 2:**
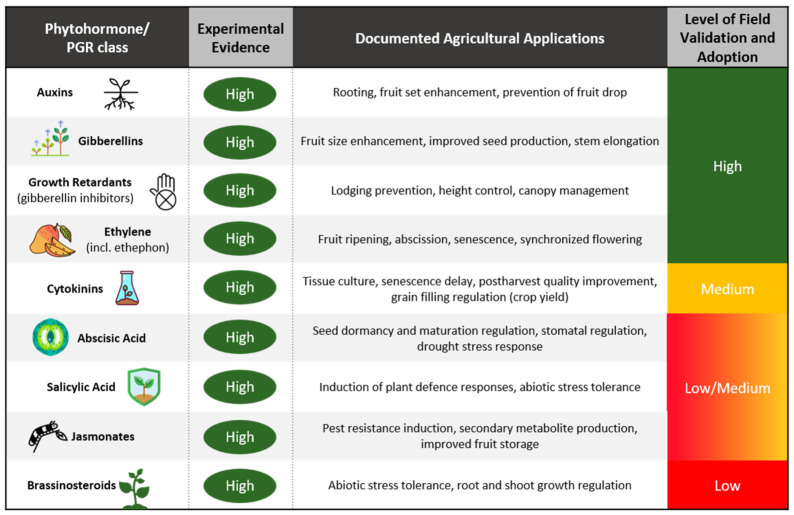
Critical summary of the phytohormones/PGRs, functional evidence, and their applications, based on previously published studies [39,40,41,42,43,44]. The figure has been created using icons from Flaticon.com, accessed on 28 April 2026.

Phytohormones are endogenously biosynthesized in plants but can also be externally applied to modulate physiological processes during critical plant development stages. Consequently, the phytohormones directly or indirectly influence plant growth, development, and crop yield. In practice, both natural hormones and their synthetic analogues (together referred to as plant growth regulators—PGRs) are used to regulate growth, development, and stress responses. Their applications enable targeted modification of agronomically important traits, including rooting, flowering, and ripening, thereby improving crop quality and yield (Figure 2).

## 3. Analyzing Phytohormones

Phytohormones have been shown to extend the shelf life of fruits and vegetables and to hold great potential as an alternative preservation technology [13,15,25,28,38,45]. Therefore, a comprehensive understanding of the molecular mechanisms and interactions of phytohormones in complex signaling networks necessitates the investigation of their concentration, diversity, and spatiotemporal distribution using advanced sensitive analytical methods [46,47]. The low levels of phytohormones in plants, along with their complex and diverse structures, make it difficult to develop improved methods for analyzing multiple phytohormones simultaneously [3].

The wide chemical diversity of the analytes complicates the development of effective sample pretreatment methods. For instance, indole-3-acetic acid (IAA), indole-3-butyric acid (IBA), 1-naphthaleneacetic acid (NAA), and gibberellic acid A_3_ (GA_3_) have acidic properties, whereas 2-isopentenyl adenine (2-IP) has basic character. Therefore, the sample extraction procedures for phytohormones have to be efficient and adapted to the wide range of chemical properties of these analytes [14].

Several publications have examined different families of phytohormones, employing common extraction techniques like classic extraction and microextraction methods to isolate these compounds from plants [18,48,49,50,51,52,53]. Both classic extraction and microextraction methods are based on liquid and solid-phase extraction [48].

Several methods have been developed, such as the solid-phase extraction (SPE) method [49], the solid-phase microextraction (SPME) [50], and the liquid–liquid extraction [51].

Algal extracts obtained through supercritical fluid extraction (SFE) were identified as a new natural source of compounds, specifically the phytohormones phenylacetic acid (PAA) and 6-benzylaminopurine (6-BA), which have the potential to promote the growth of cultivated plants [54].

Most of these methods require a high volume of solvents and involve the use of toxic organic solvents, as well as complex sample treatments such as derivatization, which is a very time-consuming procedure. Some methods include the extraction of the phytohormones for more than 12 h at low temperatures to ensure the stability of the compounds [55,56]. Other methods involved freezing or lyophilization of the samples before the extraction [57], avoiding the decomposition of the target compounds. Nevertheless, the accuracy of phytohormone quantification may be impacted by these labor-intensive, multi-step processes, which can also introduce variability and decrease reproducibility. Furthermore, techniques do not measure hormones locally but in extracts, making it difficult to directly connect the averaged measured levels to observed physiological effects.

Consequently, the development and validation of a rapid and generic sample pretreatment method applicable to diverse classes of phytohormones is still urgently required for practical applications. In the last recent years, QuEChERS (acronym of quick, easy, cheap, effective, rugged, and safe) methodology has been utilized as a sample treatment method for a wide variety of matrices before analyzing target analytes [58,59,60]. This method allows the processing of a large number of samples in a short time (quick), to be carried out in a simple way (easy), without using large quantities of solvents and reagents (cheap), completely and quantitatively extracting the amount of target analytes present in the samples (effective), being able to withstand procedural variations (rugged), and being reliable (safe), offering improved selectivity, and superior recovery rates [58].

Typical examples of sample pretreatment and analytical methods reported over the past 10 years are summarized in Table 1, which was organized based on sample type, target phytohormones, and the analytical performance of each technique, including sensitivity, selectivity, and applicability to complex plant and algal matrices.

Accurate determination of phytohormones requires developing sensitive, robust, and efficient analytical techniques. The high separation efficiency of chromatographic systems and their ability to be combined with different sensitive and selective mass spectrometer detectors make chromatography the most useful method for analyzing phytohormones [74]. Two main conventional hyphenated techniques, liquid chromatography–mass spectrometry (LC-MS) and gas chromatography–mass spectrometry (GC–MS), have been widely used to determine the levels of endogenous phytohormones [66,69,70,74,75].

Gas chromatography has the advantages of high sensitivity and very high separation efficiency. It has some limitations in the analysis of phytohormones due to the time-consuming derivatization step, which is necessary for both the electron capture detector and the mass spectrometer, as compounds in this group are mainly non-volatile [74]. Capillary electrophoresis (CE) is another established technique for analyzing phytohormones, requiring only small sample amounts and offering rapid analysis, although reproducibility issues and multi-step extraction procedures may increase experimental variability [71]. Furthermore, a lot of research measures averaged extracted phytohormones instead of localized in vivo concentrations, which makes it difficult to consistently connect hormone levels to biological outcomes like extending shelf life or promoting plant growth [76,77].

These limitations are particularly relevant for algal matrices, which contain low phytohormone levels and complex components such as polysaccharides and pigments. As a result, analytical methods developed for land plants are not always directly applicable to algae. As shown in Table 1, only a limited number of methods have been successfully adapted for macroalgae and microalgae. For macroalgae, solid-phase extraction (SPE) coupled with UPLC–MS/MS has proven effective for the simultaneous and sensitive determination of multiple phytohormones, while reducing matrix interferences [47]. Supercritical fluid extraction using CO_2_ (SFE-CO_2_) combined with HPLC-PDA has also been applied to Baltic algae, offering efficient extraction with reduced solvent use, although it requires specialized extraction instrumentation [46].

For microalgae, dispersive liquid–liquid microextraction (DLLME) combined with HPLC-FLD has been used for auxin analysis in *Chlorella vulgaris* [73]. While DLLME offers high enrichment and low solvent consumption, the limited selectivity of fluorescence detection may restrict broader phytohormone profiling. Overall, mass spectrometry-based techniques, particularly UHPLC–MS/MS combined with effective sample cleanup, appear to be the most suitable approaches for phytohormone analysis in algal systems.

Additionally, the majority of studies use laboratory or pot assays, which are unable to replicate the intricate environmental conditions found in the field accurately. The activity of phytohormones can be significantly influenced by variations in soil, microbial interactions, changes in water and nutrient levels, and other stressors, limiting the straightforward application of controlled studies to real-world agricultural scenarios [78,79].

The most frequently used method for phytohormone analysis is high-performance liquid chromatography combined with different detectors, such as ultraviolet (UV) [50,64,65], diode array [46,63,65,68], and fluorescence detector (FLD) [61,73]. Due to its high sensitivity, selectivity, and good linearity, HPLC coupled to MS/MS allows reliable quantification of compounds at trace levels. Significant progress has been made in recent years in the simultaneous determination of numerous phytohormones using liquid chromatography–tandem mass spectrometry (LC-MS/MS) [49,58,66,72,80].

The application of ultra-high-performance liquid chromatography(UHPLC) improves resolution and speed compared with HPLC, and shows high sensitivity to plant hormones [47,51,59,60,67,75].

UHPLC-MS/MS offers several key advantages in phytohormone analysis. This advanced technique offers unparalleled sensitivity and accuracy, making it a valuable tool for understanding the intricate mechanisms of plant growth and development. Therefore, highlighting the importance of this technique can greatly improve the quality and comprehensiveness of phytohormone analysis. To confirm the biological significance and practical applications of phytohormones, future research should focus on developing standardized, high-throughput in vivo techniques and incorporating field trials.

Overall, phytohormone detection methods differ in sensitivity, selectivity, cost, and applicability. LC-MS/MS and UHPLC-MS/MS are the most powerful techniques, offering very high sensitivity, selectivity, and accurate quantification even at trace levels, making them suitable for complex biological matrices. However, they are expensive, require advanced technical expertise, and involve complex sample preparation. In comparison, HPLC coupled with UV/DAD or FLD detection is more accessible and cost-effective but has lower sensitivity and a higher risk of interference, especially in complex samples. GC-MS provides good resolution for volatile or derivatized compounds but is less suitable for thermally unstable phytohormones. Techniques such as SPE, SPME, and CE are mainly used as sample preparation or complementary methods. They are fast and economical but generally insufficient for comprehensive multi-phytohormone profiling.

Therefore, although advanced MS-based techniques dominate current research, there is still a need for standardized, rapid, and high-throughput approaches that balance sensitivity, cost, and practicality, particularly for algal and plant matrices.

## 4. Exploring Algae as a Source of Phytohormones

Phytohormones are vital compounds that support plant growth and development processes. Due to their low plant concentrations, they can also be sourced from alternatives like algae. These compounds that stimulate algal growth increase crop yield and enhance plant quality, while also helping plants withstand various stresses such as pests, diseases, and environmental factors. Plant hormones can be derived from algae or synthesized externally [45].

**Figure 3:**
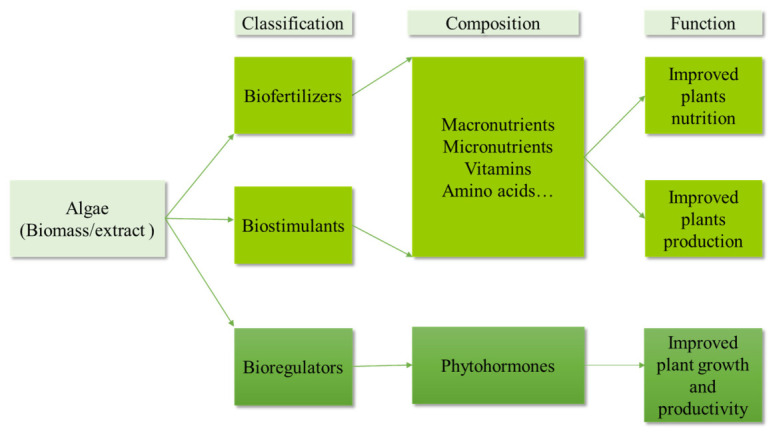
Classification of major functions of algae extracts and biomass in crop cultivation.

Figure 3 illustrates the relationship between phytohormones and algae, indicating how algae-derived substances or compounds can influence the production or activity of plant hormones.

### 4.1. Phytohormones Within Macroalgae

In marine ecosystems, macroalgae play a significant role among diverse species. Each group of these marine algae contains phytohormones like auxins, gibberellins, cytokinins, abscisic acid, and ethylene [81]. These phytohormones offer benefits across various industries, including agriculture, food and nutraceuticals, pharmaceuticals, and biofuels [45,82].

Species such as *Ascophyllum nodosum* (L.) Le Jolis, *Macrocystis pyrifera* (L.) Ag., *Laminaria digitata* (Hudson) Lam., *Durvillaea potatorum* (Lab.) Ares. from the brown algae, *Kappaphycus alvarezii* (Doty) Liao from the red algae, or *Ulva* species from the green algae genus are among those utilized in plant applications related to plant growth and cultivation [46,83]. The main plant growth regulators, such as indole-3-acetic acid, abscisic acid, gibberellic acid, zeatin, kinetin, and 6-benzylaminopurine, were detected in nine seaweed varieties [47].

Endogenous phytohormones were quantified in 11 species of red algae collected from the Brazilian coast. Indole-3-acetic acid and indole-3-acetamide (IAM) were the primary auxins identified across all species. ABA was detected in all species except for *Hypnea nigrescens* Greville ex Agardh, and no ABA conjugates were found in any species. Cytokinins, auxins, and ABA were prevalent components in red algae, marking the first discovery of ABA in Rhodophyta [84].

Nine plant hormones were identified in seaweed extracts from a mixture of Baltic algae, *Cladophora glomerata* (L.) Kütz., and *Arthrospira* spp. These included IAA, IBA, phenylacetic acid (PAA), naphthylacetic acid (NAA), *trans*-zeatin (TZ), kinetin (KA), isopentenyladenine (IA), 6-benzylaminopurine (6-BA), and ABA [46].

### 4.2. Phytohormones Within Microalgae

Phytohormones, widely present in microalgae as chemical messengers, have metabolisms that are not as well understood as those in higher plants.

The primary challenge arises from the low content of phytohormones in microalgae cells, coupled with the complexity of analysis and detection methods. Very little is known about the regulatory role of phytohormones in microalgae [23].

Microalgae and cyanobacteria widely produced at an industrial scale, including chlorophytes such *as Chlorella vulgaris* and cyanobacterial genera such as *Arthrospira* and *Nostoc*, have been employed as sources for plant biostimulant production [45,85]. Phytohormones such as auxins and cytokinins (e.g.,indole-3-acetic acid and 6-benzyladenine) are endogenously produced by several cyanobacteria (e.g., *Nostoc muscorum*) and green microalgae (e.g., *Chlorella vulgaris*), and have been successfully isolated and quantified [45]. In addition, recent genetic evidence demonstrates that the model alga *Chlamydomonas reinhardtii* can produce auxin via the LAO1-mediated pathway, expanding our understanding of algal phytohormone biosynthesis [86].

## 5. Exploring Algae as Biofertilizers, Biostimulants, and/or Bioregulators in Agriculture

In Africa, America, and Asia, algae have been used for millennia for medicinal, cosmetic, and nutritional purposes. These applications include a wide range of algal forms, from microalgae to macroalgae and from unicellular to multicellular species [87]. In the European Union, however, only a limited number of microalgae are currently authorized for human consumption, notably *Chlorella vulgaris* and *Arthrospira* spp., in accordance with the EU Novel Food Regulation (Regulation (EU) 2015/2283) [88].

The agricultural use of algae dates back thousands of years. In the 20th century, farmers worldwide began to show interest in products derived from algal extracts [89].

**Figure 4:**
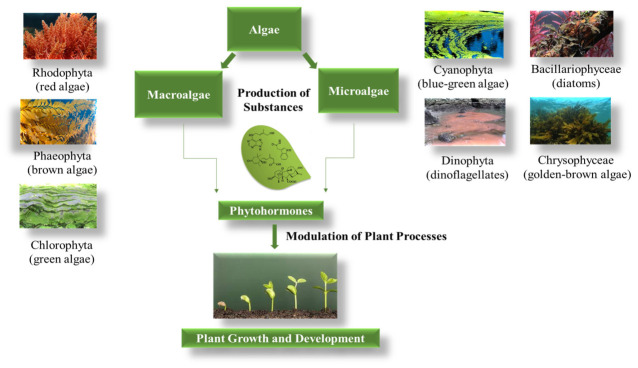
Influence of algae-derived phytohormones on plant growth.

Algae are utilized in agriculture as biofertilizers and biostimulants, such as the model microalgae *Chlamydomonas* [90], because they contain essential macronutrients, micronutrients, vitamins, and amino acids. Biofertilizers improve plant nutrition by increasing the availability of nutrients in the soil. Biostimulants enhance plant growth, development, and stress tolerance through bioactive compounds, rather than by supplying nutrients. Bioregulators, which are often phytohormones or growth regulators, directly regulate plant physiological processes even at low concentrations (Figure 4) [91].

However, significant variation exists among algal species in their functional effectiveness in agriculture. The effectiveness of algae in agriculture varies significantly among species. For example, brown seaweeds such as *Ascophyllum nodosum* have been widely reported to strongly enhance plant growth, stress tolerance, and yield due to their high levels of plant growth regulators, polysaccharides, and micronutrients [92]. In contrast, *Sargassum* species (macroalgae) are often more effective at improving soil fertility and nutrient availability, but their effects on direct stimulation of plant growth are sometimes less consistent. Microalgae such as *Chlorella* and *Spirulina* are rich in proteins, amino acids, and bioactive compounds, and are particularly effective as biofertilizers and biostimulants. However, their application may require additional processing to achieve stable field performance [93]. Overall, these differences show that algal species cannot be considered equally effective, and their selection should depend on the specific agricultural goal, such as plant growth promotion, soil improvement, or stress resistance.

### 5.1. Algae as Biofertilizers

Bio-fertilization is an eco-friendly agricultural approach that uses biofertilizers to improve soil nutrition and support crop productivity [94]. A biofertilizer is a product containing living microorganisms that exert direct or indirect beneficial effects on plant growth and yield through various mechanisms [95].

Biofertilizers, such as algal-based products, contribute to long-term soil fertility and are essential for meeting global food demands, primarily by improving soil nutrient availability and microbial activity. Microalgae and cyanobacteria have been used as biofertilizers or organic fertilizers to enhance the biological and chemical characteristics of the soil [91]. Their contribution mainly involves nutrient enrichment and soil conditioning [96].

The University of Texas’s Algae Processing Program indicated that microalgae could significantly impact the future of agriculture [97]. *Chlorella vulgaris* (microalga) and *Arthrospira platensis* Gom. (microalga) were applied as a biofertilizer in rice cultivation. Increased rice yield from 7% to 20.9% were observed, respectively, making microalgae a viable “green” alternative to chemical fertilizers [98].

Using a suspension of the microalga *Chlorella* sp. caused an increase in germination rate for wheat, barley, and maize seeds [99]. Similarly, the supercritical fluid extracts from biomass positively impact the germination of cress and winter wheat seeds [53].

Cyanobacteria have a wide geographical distribution and present high resilience to environmental stress [100]. They share certain similarities with bacteria and plants and are well adapted to the wet environment of rice paddies, where they are commonly applied in the process of ‘algalization’ [101].

The use of cyanobacteria resulted in crop yield and quality comparable to or better than that achieved using only chemical fertilizer. This is largely attributed to their role in nutrient supply, particularly nitrogen fixation.

Special focus was given to cyanobacterial biomass or extracts because of their clear potential as a valuable source of vital nutrients and metabolites with various biological activities that can greatly enhance the productivity of crops [102]. Seeds treated with microalgae showed increased lateral roots, enhancing plants’ capacity to absorb water and nutrients [103]. The consortia between cyanobacteria and green algae can boost wheat growth and yield, enhance microbial activity, and raise levels of organic carbon, macro-, and micronutrients in the soil [104]. These studies involve diverse plant taxa, but *Chlamydomonas reinhardtii* (microalga) has been used as a reference model organism to understand and optimize algal–microbial consortia by examining their molecular and physiological mechanisms [90].

The unicellular microalgae *Nannochloropsis*, whose biomass was harvested and washed to remove residual salts before application, have been shown to enhance both the sugar and carotenoid levels in tomatoes [103].

There is now also a growing interest in utilizing macroalgae for their potential as an organic fertilizer or soil conditioner. Since the 18th century, these seaweeds have been used as a fertilizer, but only in coastal regions [97].

Seaweed, or ocean macroalgae, has an important role in boosting soil humus content. While seaweeds are unable to fix atmospheric nitrogen, a process that is limited to specific microalgal groups like nitrogen-fixing cyanobacteria, recent studies have highlighted the biotechnological potential of microalgal systems, particularly when associated with nitrogen-fixing bacteria [105]. Seaweeds nevertheless remain a valuable source of growth regulators and essential micro- and macronutrients. Seaweed-based fertilizers are designed to improve seed germination, promote deeper root growth, increase nutrient uptake, and enhance crop yield in the treated plants [97].

Such seaweeds as *Sargassum* spp. and *Gracilaria* spp. are commonly utilized as fertilizers to enhance the growth of coconut and paddy crops in India [106].

Seaweed is being used directly in agricultural fields either as dried powder in the form of fertilizers or as liquid extracts (SLE) [97,107]. Natural fertilizers derived from algae are considered to be more effective than farmyard manure and chemical fertilizers. Algal-based fertilizers are being promoted as a new approach to address sustainability challenges in agriculture due to their ease of use, affordability, longer shelf life, enhanced aeration, and ability to increase plant resistance to various diseases, pests, insects, nematodes, and environmental stressors like drought, frost, and salt [91]. Seaweed extract speeds up seed germination rates even when applied in lower concentrations. Commercially accessible SLEs are mainly derived from brown seaweeds and exhibit viscosity, color, scent, and pH differences [91].

### 5.2. Algae as Biostimulants

Plant growth and development depend on a good growing environment, including healthy soil, nutrient availability, and protection from pests and other stresses. These requirements can be fulfilled either through natural means or by artificial provisioning.

Biostimulants, also known as ‘metabolic enhancers’ [108], are substances aside from fertilizers that improve crop yield by acting directly on the plant, regardless of their nutrient levels. These substances help enhance plants’ metabolism, thus improving their growth by boosting respiration, photosynthesis, nucleic acid synthesis, and ion uptake, and imparting resistance to various biotic and abiotic stresses [109,110].

According to the last European regulation on fertilizers, EU2019/1009, a biostimulant is a product of natural origin that “stimulates plant nutrition processes independently of the product’s nutrient content with the sole aim of improving the nutrient use efficiency, tolerance to abiotic stress, quality traits, and availability of confined nutrients in soil or rhizosphere” [111].

The use of algae as plant biostimulants has become more significant in recent years for enhancing crop growth and development, alongside other methods [110]. Brown macroalgae, or Phaeophyceae (Chromista, Ochrophyta), the second-largest group of macroscopic algae, with around 2100 identified species, are among the most promising classes [112]. The use of brown macroalgae extract (BME) is to boost crop yield, promote plant health and quality, and enhance soil health and fertility [113].

Brown macroalgae grow naturally in the ocean, so they do not compete for agricultural land and need no irrigation or fertilizers to flourish [114]. Hence, BMEs are quickly progressing in the biostimulant industry, with the introduction of many commercial products [115].

Microalgae contain various bio-stimulatory compounds, including phenolic compounds, terpenoids, polysaccharides, and amino acids [85,102]. Extracts and metabolites derived from various microalgae species like *Chlorella* spp., *Arthrospira platensis*, *Acutodesmus* spp., *Scenedesmus* spp., *Dunaliella* spp., *Calothrix elenkini* Koss., etc., are frequently employed as biostimulants [85,116].

For instance, phycocyanin extract from *Arthrospira platensis* enhanced the germination rate and increased the biomass yield of tomato (*Solanum lycopersicum* L.) crops [115]. The consortia of *Chlorella* spp., *Scenedesmus* spp., *Chlorococcum* spp., *and Micractinium* spp. enhanced the nutritional content of spinach (*Spinacia oleraceae* L.) seeds and boosted their biomass production [116], indicating improved physiological efficiency rather than repeated general growth effects. Accordingly, *Chlamydomonas reinhardtii* serves as a model system for exploring the cellular and molecular mechanisms governing algal–microbial interactions that contribute to the positive outcomes of applied algal consortia [117].

Under field conditions, microalgae may also interact synergistically with plant growth-promoting bacteria (PGPB), further enhancing their biostimulant effects. For example, associations between microalgae and *Methylobacterium* spp. have been reported to stimulate plant growth through mutualistic metabolic exchanges, such as proline–glycerol cycling, which supports both microbial partners and promotes plant performance [118]. These interactions can enhance nutrient use efficiency, stress tolerance, and overall plant growth, highlighting that the biostimulant activity of microalgae is not solely attributed to their bioactive compounds, but also to their capacity to modulate beneficial microbial communities in the plant–soil environment [118].

Foliar spray application of *Chlorella vulgaris* extract had a significant impact on lettuce (*Lactuca sativa* L.) crops by increasing yields, boosting leaf phytochemical levels, enhancing enzymatic activity, and improving tolerance to both biotic/abiotic stress [119]. Spraying exopolysaccharides from the microalgae *Dunaliella salina* (Dunal) Teod. [120] and *Scenedesmus subspicatus* Chodat [121] directly on the leaves led to increased biomass and harvest yield of tomato (*Solanum lycopersicum* L.) and onion (*Allium cepa* L.), respectively [120,121]. In addition to boosting crop yield, the soil quality for bell pepper (*Capsicum annuum* L.) and eggplant (*Solanum melongena* L.) crops was also improved using *Navicula* spp. extract [122].

Sustainable agriculture practices promote eco-friendly plant growth stimulators to meet the rising need for organically grown crops and to create innovative bio-based options. Although seaweed extracts and cyanobacteria have been utilized in agriculture for a long time [123] the introduction of novel methods, such as the omics approach in microalgae biotechnology, brings about fresh possibilities for improving plant growth. Certain important research and development pathways are necessary for the effectiveness of algae-based technologies in enhancing crop productivity [102]. In this scenario, it is crucial to explore a variety of algal species, extraction methods, plant species, and plant growth conditions to understand how algal extracts impact plant growth, development, and resilience to environmental stressors [124]. Various products with diverse chemical compositions and formulations help stimulate plants and soil, reducing abiotic stresses like drought, frost, and salt. However, the exact connection between the chemical makeup of the extract and its resulting impact has not yet been fully understood, with a potentially significant role of molecular synergy. Hence, advances in genomic methods and thorough analysis of micro and macroalgae may lead to a deeper understanding of plant processes, enabling the optimization of extracts for enhanced effectiveness in the future [125].

### 5.3. Algae as Bioregulators

Currently, algae-based agricultural production methods are utilized in both organic and conventional farming, where they act through hormonal regulation mechanisms.

Algae extracts have the properties of bioregulators, with phytohormones or growth regulators, offering an innovative approach to sustainable agriculture by improving nutrient uptake, crop yield, and resilience to biotic and abiotic stresses [126]. These plant hormones fall into two categories: Plant Growth Promoters and Plant Growth Inhibitors. They include auxins, ABA, gibberellins (GA), cytokinin (CK), ethylene (ET), SA, JA, and brassinosteroids (BR). These hormones were discovered in various algal species with levels similar to those in higher plants. Consequently, seaweed and microalgae extracts have been utilized commercially to stimulate growth or regulate crops [85,124,125]. Phytohormones from macroalgae act as external growth regulators that impact the ability to withstand a variety of abiotic and biotic stress factors [45].

Seaweed extracts known as seaweed liquid fertilizer (SLF) and/or seaweed liquid biostimulant (SLB) are usually preferred due to trace elements and metabolites related to plants’ growth-promoting hormones. These seaweed extracts are employed in farming to reduce the need for harmful agrochemicals and help protect the environment. Integrating them into common agricultural practices can boost crop production in eco-friendly ways [45].

The impact of SLFs from *Sargassum* spp. on the black gram (*Vigna mungo* L.) under saline stress indicated that the use of a 10% SLF concentration was the most successful treatment in enhancing the morphology, chlorophyll pigments, and protein content of black gram plants experiencing salt stress [127]. Plants like *Sorghum bicolor* (L.) Moench and *Pennisetum glaucum* (L.) R. Br. treated with 0.50% seaweed liquid fertilizer, such as *Sargassum cinereum* J. Agardh and *Ulva intestinalis* L., had increased protein and total chlorophyll levels, as well as earlier flowering and fruiting compared to untreated plants [128].

*Vigna radiata* (L.) Wilczek displayed superior growth, proximate compositions, mineral contents, and fatty- and amino acid contents when treated with 20% SLB from *Halimeda opuntia* (L.) Lam [129].

The enhancement of plant growth by applying macroalgal extracts has been observed in diverse crops and systems such as *Comanthera mucugensis* (Giul.) L.R.Parra & Giul. in vitro [130], pepper in the greenhouse [131], and Solanaceae crops in greenhouses and fields [132]. In saline environments, the application of extracts from different macroalgae increased the tolerance of rapeseed and wheat plants to abiotic stress. This effect is attributed to indole acetic acid, indole butyric acid, gibberellic acid, cytokinins, total carbohydrates, and phenolic compounds in the extracts [133].

Phytohormones found in microalgae and cyanobacteria have been proven to enhance plant growth [124,134]. The availability of biomass and extracts from microalgae and cyanobacteria commercially is due to their positive influence on the progress of sustainable agriculture [135].

Elakbawy et al. (2022) detected and measured the levels of naturally occurring plant hormones, auxins and cytokinins (IAA and BA), in cyanobacteria, showing that *Aulosira fertilissima* Ghose positively impacted the growth of rice (*Oryza sativa* L.) [136], where the growth of rice seedlings was boosted by root-promoting hormones such as auxins, cytokinins, and gibberellic acid [137]. Using algal phytohormones or plant growth regulators could offer new possibilities for phytohormone utilization in agriculture, expanding the potential for macro and microalgae utilization [138]. This is especially relevant as certain algal species from the *Ascophyllum* and *Laminaria* genera produce significant amounts of ABA, which can be extracted to improve plant growth under stressful conditions [85]. Algal auxins are utilized to boost the sprouting and development of the *Aegle marmelos* (L.) Corrêa medicinal plant, as well as the rooting of different fig cuttings [139].

Algal cytokinins can also enhance the growth of cotton seedlings by 10% in drought conditions [140]. Therefore, algal cytokinins are used as plant growth stimulants to promote sustainable agriculture [141]. It serves as a defense against diseases in tomatoes, including antibacterial, antifungal, and antiviral properties, as well as providing fertilization, biostimulation, and antioxidants [89,142].

Despite the promising role of algae-derived products in agriculture, their field-scale application and commercialization remain limited by several practical constraints. Variability in algal species composition, extraction techniques, and environmental conditions can affect product consistency and performance under field conditions. Moreover, scalability, cost-effectiveness, and storage stability represent additional challenges for industrial production and market adoption. Consequently, further research is required to develop standardized and economically viable production systems supported by extensive field validation.

## 6. Conclusions

Phytohormones play a vital role in the growth and development of plants as well as their biotic and abiotic stress responses. Therefore, they are crucial for successful crop production. Since they are present in small amounts within plants, they can be supplemented from alternative sources like algae and have various uses. These substances, which promote algal growth, were also found to enhance the biomass and quality of cropped plants.

This review highlights the application of algae as biofertilizers, biostimulants, and bioregulators in crop production, with particular emphasis on naturally occurring phytohormones. However, despite promising results, clear gaps remain regarding the standardization of algal extraction methods, dose response relationships, and the understanding of synergistic effects among algal metabolites under field conditions. Most available studies are based on laboratory or greenhouse experiments, limiting the direct translation of these findings to large-scale agriculture.

Future studies should concentrate on identifying the main bioactive substances causing plant reactions, field-based validation, and repeatable formulation techniques. The practical integration of algae-based products into sustainable agricultural practices will depend on establishing consistent performance, scalability, and regulatory compliance.

## Figures and Tables

**Table 1:** Extraction and detection methods of plant hormones in different plant matrices grouped by organism/tissue type.

Organism/Tissue Type	Extraction/ Purification Method	Detection Method	Analyte(s)	Plant Matrix/Species	References
Fruits	DLLME	HPLC-FLD	IPA, NAA, IAA, IBA, JA, OPDA, GA	Grapes, cherry, nectarine, apple, litchi	[61]
		LC–ITMS	SA, ABA	Peach	[62]
	SPE	HPLC–DAD	GA, Z, PBZ, 4-FPA, 4-CPA, IAA, IBA, 6-BA, ABA, NAA, CPPU, 2,4-D, 2,4,5-T	Kiwi, strawberry, bean sprout, green pepper	[63]
	SPME	HPLC-UV	NAA, 2,4-D, IAA, SA	Tomato, grape juice	[64]
		LC–UV/DAD	SA	Blueberries, kiwi, tangerines, lemons, oranges, fruit juice	[65]
	SPE/LC-MS/MS	LC-MS/MS	Cytokinin, ABA, IAA, GA7, SA	Strawberry	[66]
Vascular plants (leaves, whole plants, sprouts, flowers, seeds)	SPE	LC-MS/MS	ETH, Auxin, Cytokinin, ABA, JA, SA	*Arabidopsis*	[49]
	SPE	Triple Quad LC-MS/MS	Aux, GA, JA, ABA, SA	Rice	[67]
	SPME	HPLC-UV	ABA, GA3, IAA	Cucumber, tomato, date	[50]
	DSPE	HPLC-DAD	IAA, IBA, 1-NAA, 2-NAA, ABA	Mung bean sprouts	[68]
	LLE	UHPLC-ESI-MS/MS	GAs	*A. thaliana* flower	[51]
		GC-MS	Auxins, SAs, JAs, ABA	Sweet orange (*Citrus sinensis* (L.) *Osbeck*)	[69]
		GC-MS	ABA, IAA, JA, SA	Leaves of *M. truncatula*	[70]
		CE-DAD	ABA, IAA, GA	Plant and seaweed extracts	[71]
	QuEChERS	HPLC-MS/MS	ZT, ZR, IAA, ABA, GA3	Cotton	[58]
		UHPLC-MS/MS	2,4-D, GA, NAA, NA	Cucumber, orange, tomato, watermelon, zucchini	[59]
		UHPLC-MS/MS	BA, GA, IAA, NAA, NA, 2,4-D	Courgette	[60]
	EME	LC-MS/MS	JA, ABA, SA, BA, GA3, GA4	Hamlin trees (*Citrus sinensis*)	[72]
Macroalgae/Seaweeds	SPE	UPLC-MS/MS	IA, ABA, GA, Z, KA, BAP	Seaweeds	[47]
	SFE-CO_2_	HPLC-PDA	IAA, IBA, PAA, NAA, TZ, KA, IA, 6-BA, ABA	Baltic algae	[46]
Microalgae	DLLME	HPLC-FLD	Auxins	*Chlorella vulgaris* and *Duranta* young leaves	[73]

Table legend: DLLME (dispersive liquid–liquid microextraction), SPE (solid-phase extraction), SPME (solid-phase microextraction), DSPE (dispersive solid-phase extraction), LLE (liquid–liquid extraction), EME (electromembrane extraction), SFE-CO_2_ (supercritical fluid extraction with carbon dioxide), HPLC (high-performance liquid chromatography), UHPLC (ultra-high-performance liquid chromatography), UPLC (ultra-performance liquid chromatography), LC-MS/MS (liquid chromatography–tandem mass spectrometry), GC-MS (gas chromatography–mass spectrometry), CE (capillary electrophoresis), FLD (fluorescence detector), DAD (diode array detector), PDA (photodiode array detector), ESI (electrospray ionization), ITMS (ion trap mass spectrometry), and MS/MS (tandem mass spectrometry).

## Data Availability

No new data were created or analyzed in this study.

## Abbreviations

ABA	Abscisic Acid
6-BA	6-benzylaminopurine
BRs	Brassinosteroids
CE	Capillary electrophoresis
4-CPA	4-chlorophenoxyacetic acid
CPPU	Forchlorfenuron
CTK	Cytokinin
DAD	Diode array detector
2,4-D	2,4-Dichlorophenoxyacetic acid
DLLME	Dispersive liquid–liquid microextraction
DSPE	Dispersive Solid-Phase Extraction
EME	Electromembrane extraction
ESI	Electrospray ionization
ETH	Ethylene
FLD	Fluorescence detector
4-FPA	4-fluorophenoxyacetic acid
GA	Gibberellins
GA_3_	Gibberellic acid (A3)
GA_4_	Gibberellic acid (A4)
GC-MS	Gas chromatography–mass spectrometry
HPLC	High-performance liquid chromatography
IAA	Indole-3-acetic acid
IA	Isopentenyladenine
IBA	Indole-3-butyric acid
IPA	Indole-3-propionic acid
ITMS	Ion trap mass spectrometry
JA	Jasmonate
KA	Kinetin
LC-MS/MS	Liquid Chromatography coupled to tandem Mass Spectrometry
LLE	Liquid–liquid extraction
NA	Naphthylacetamide
NAA	1-Naphthaleneacetic acid
OPDA	12-oxo-phytodienoic acid
PAA	Phenylacetic acid
PBZ	Paclobutrazol
PDA	Photodiode array
PGRs	Plant Growth Regulators
SA	Salicylic Acid
SFE-CO_2_	Carbon Dioxide Supercritical Extraction
SPE	Solid-phase extraction
SPME	Solid-phase microextraction
2,4,5-T	2,4,5-trichlorophenoxyacetic acid
TZ	Trans-Zeatin
UPLC	Ultra-performance liquid chromatography
UV	Ultraviolet
ZR	Zeatin riboside
